# Exposure of the Opportunistic Marine Pathogen *Photobacterium damselae* subsp. *damselae* to Human Body Temperature Is a Stressful Condition That Shapes the Transcriptome, Viability, Cell Morphology, and Virulence

**DOI:** 10.3389/fmicb.2020.01771

**Published:** 2020-07-24

**Authors:** Xosé M. Matanza, Carlos R. Osorio

**Affiliations:** Departamento de Microbioloxía e Parasitoloxía, Instituto de Acuicultura, Universidade de Santiago de Compostela, Santiago de Compostela, Spain

**Keywords:** *Photobacterium damselae*, zoonosis, temperature, transcriptome, hemolysin, heat-shock response

## Abstract

*Photobacterium damselae* subsp. *damselae* (*Pdd*), an important pathogen for marine animals, is also an opportunistic human pathogen that can cause fatal necrotizing fasciitis. The regulatory changes triggered by the temperature shift experienced by this marine pathogen upon entering the human body, are completely unknown. Here we report an RNA-seq approach combined with phenotypical assays to study the response of *Pdd* to cultivation at 37°C in comparison to 25°C. We found that cultivation of a *Pdd* highly virulent strain for fish and mice, RM-71, at 37°C, initially enhanced bacterial growth in comparison to 25°C as evidenced by the increase in optical density. However, cells were found to undergo a progressive loss of viability after 6 h cultivation at 37°C, and no viable cells could be detected from 30 h cultures at 37°C. In contrast, at 25°C, viable cell counts achieved the highest values at 30 h cultivation. Cells grown at 25°C showed normal rod morphology by scanning electron microscopy analysis whereas cells grown at 37°C exhibited chain-like structures and aberrant long shapes suggesting a defect in daughter cell separation and in septum formation. Cells grown at 37°C also exhibited reduced tolerance to benzylpenicillin. Using a RNA-seq approach we discovered that growth at 37°C triggered a heat-shock response, whereas genes involved in motility and virulence were repressed including iron acquisition systems, the type two secretion system, and damselysin toxin, a major virulence factor of *Pdd*. Human isolates did not exhibit advantage growing at 37°C compared to fish isolates, and comparative genomics did not reveal gene markers specific of human isolates, suggesting that any *Pdd* genotype existing in the marine environment might potentially cause disease in humans. Altogether, these data indicate that the potential of *Pdd* to cause disease in humans is an accidental condition rather than a selected trait, and that human body temperature constitutes a stressful condition for *Pdd.* This study provides the first transcriptome profile of *Pdd* exposed at human body temperature, and unveils a number of candidate molecular targets for prevention and control of human infections caused by this pathogen.

## Introduction

*Photobacterium damselae* subsp. *damselae* (hereafter *Pdd*) is a bacterium of the family *Vibrionaceae* pathogenic for a broad range of hosts including marine animals and humans (Osorio et al., 2018). It represents a major threat for marine fish aquaculture worldwide, and disease outbreaks in fish farms are typically preceded by increases in sea water temperature during summer months (Fouz et al., 1992; Pedersen et al., 2009; Eissa et al., 2018; Yu et al., 2019). In humans, *Pdd* can cause severe wound infections and necrotizing fasciitis that may evolve into a fatal outcome despite prompt antibiotic administration (Morris et al., 1982; Clarridge and Zighelboim-Daum, 1985; Yuen et al., 1993; Asato and Kanaya, 2004; Yamane et al., 2004; Hundenborn et al., 2013; Akram et al., 2015; Alhemairi et al., 2015). Notably, some authors recommend to surgically debride and to amputate at an early stage of the infection to save lives of patients (Goodell et al., 2004). Underlying diseases (as diabetes, liver disorders and immunodeficiency among others) may accompany infection (Knight-Madden et al., 2005; Nakamura et al., 2008; Kim et al., 2009), but they are not a prerequisite for the development of disease since apparently healthy individuals are also susceptible (Morris et al., 1982; Barber and Swygert, 2000; Yamane et al., 2004). Human infections by *Pdd* originate through exposure of subcutaneous tissue to the marine environment or through wounds inflicted during fish handling. *Pdd* is considered one of the main zoonotic pathogens acquired topically from fish (Lehane and Rawlin, 2000; Austin, 2010), and the majority of human cases concentrate in coastal areas of the United States of America, Japan and Australia, during the warm season.

The major reported virulence factors of *Pdd* are cytotoxins with hemolytic activity (Osorio et al., 2018). The virulence plasmid pPHDD1 encodes the cytotoxins damselysin (Dly) and phobalysin P (PhlyP) (Rivas et al., 2011), and two additional cytotoxins, phobalysin C (PhlyC) and phospholipase PlpV are encoded within chromosome I (Rivas et al., 2013; Vences et al., 2017). These virulence factors are secreted via the type II secretion system (Rivas et al., 2015a; Vences et al., 2017). Recently, it was reported that the two-component regulatory system RstAB regulates transcription of the genes encoding Dly, PhlyP, and PhlyC toxins, and consequently its inactivation strongly impairs virulence (Terceti et al., 2017, 2019).

Despite the aggressive outcome of *Pdd* infections in humans, nothing is known about how this bacterium responds to the temperature shift that it encounters in its transition from warm sea waters to invading a human host. In order to shed light on this, in the current work we compared the transcriptomes of *Pdd* at 25 and 37°C, mimicking the environmental vs. the host temperature conditions. In addition, the genome sequences of two human isolates of *Pdd* are reported for the first time, and a comparative genomics analysis between human and fish strains is presented. Growth assays, viability tests and cell morphology studies, together with the transcriptomics data, all suggest that growth at 37°C represents a stressful condition and not an evolutionarily selected trait for *Pdd*. Overall, these data are expected to contribute to the investigation of novel approaches for prevention and for less invasive control of human infections caused by *Pdd* in a global warming scenario.

## Materials and Methods

### Culture Conditions, Viability Assays, and Fatty Acid Methyl Ester (FAME) Analysis

*Pdd* strains were grown at 25 or 37°C on tryptic soy agar (TSA) or in tryptic soy broth (TSB) supplemented with NaCl up to 1% (TSA-1 and TSB-1, respectively). For growth curves, three replicates for each temperature of the assay (25 and 37°C) were grown in TSB-1 until obtaining exponentially growing precultures (OD_600_: 0.3). Then, 1:100 dilutions of each preculture were grown in 100 μl of TSB-1 in 96 well plates and the optical density (OD_600_) was measured using the spectrophotometer Epoch2 microplate reader (Biotek). For drop-plating viability assays, three independent cultures were grown at each temperature using the spectrophotometer Epoch2 microplate reader under the same conditions used for growth curve analysis, and 5 μl aliquots of 10-fold dilutions were drop-plated in TSA-1 plates after 6, 12, 24, 30, 36, and 48 h of incubation, and plates were incubated at 25°C for 24 h. FAME analysis was performed by extracting and preparing fatty acid methyl esters from 24 h *Pdd* RM-71 cultures on TSA-1 at 25 or 37°C and using the MIDI Sherlock^®^ Microbial Identification System (MIDI, Inc., United States), following manufacturer’s recommendations.

### Genome Sequencing and Comparative Genomics

Draft genome sequences of human isolates CDC-2227-81 (Kreger, 1984) and 80077637 (Hundenborn et al., 2013) were obtained using an Illumina platform as previously described (Abushattal et al., 2019) and annotated using RAST tool (Aziz et al., 2008). Sequence similarity analysis by comparison of orthologous fragments between pairs of genomes was conducted with OrthoAni (Lee et al., 2016). Core genome and unique genes were calculated using RAST (Aziz et al., 2008). Genomic BLAST file of *Photobacterium damselae* subsp. *damselae* strains 80077637, CDC-2227-81, RM-71, CIP102761, A-162, and LD-07 was downloaded from NCBI^1^, and the dendrogram was visualized by Interactive Tree of Life (iTOL v5) (Letunic and Bork, 2019). The draft genome sequences obtained in this study have been deposited at GenBank/EMBL/DDBJ under accession number VZUQ00000000 (CDC-2227-81) and WAEO00000000 (80077637).

### Scanning Electron Microscopy

Exponentially growing cultures of *Pdd* RM-71 in TSB-1 at each temperature were stopped when they reached an OD_600_ of 0.55, cells were carefully pelleted down by centrifugation (4,000 *g*) and fixed for 3 h at 4°C in 4% paraformaldehyde and 2% glutaraldehyde in 0.1 M phosphate buffer, pH 7.4, and postfixed for 1.5 h in 1% osmium tetroxide in the same buffer. Samples were washed three times in dH_2_O, dehydrated using a series of graded ethyl alcohols, chemically dried using HMDS (hexamethyldisilazane) (Sigma), sputter-coated with iridium, and viewed and photographed in an Ultra Plus ZEISS scanning electron microscope.

### *E*-test Assay

To determine the susceptibility to benzylpenicillin at the two assayed temperatures, *Pdd* strain RM-71 was grown at a temperature of 25°C in 10 ml TSB-1 in 100 ml flasks, and collected when cultures reached a sharp optical density at 600 nm (OD_600_) of 0.5. Aliquots of 100 μl were spread onto TSA-1 plates in the presence of *E*-test gradient antibiotic strips (bioMérieux), and incubated for 24 h at 25°C and 37°C.

### RNA-Seq

For RNA-seq, three independent precultures of RM-71 strain for each temperature condition were started and grown until an OD_600_ of 0.3. Then, 1:100 dilutions of each preculture were grown in 10 ml of TSB-1 in 100 ml flasks until they reached a sharp OD_600_ of 0.55. Cells were immediately treated with RNAprotect Bacteria Reagent (Qiagen) for stabilization of RNA following manufacturer’s instructions. Pelleted cells were then carefully resuspended in 100 μl TE buffer (30 mM Tris⋅Cl, 1 mM EDTA, pH 8.0), adding 10 μl lysozyme (15 mg/ml) (Sigma Aldrich) and 15 μl Proteinase K (20 mg/ml) (Qiagen). RNA was extracted with RNeasy Mini Kit (Qiagen), and DNase I treatment was performed using the on-column kit RNase-free DNase (Qiagen). The quality and the quantity of the total RNA was determined using a Bioanalyzer 2100 (RNA 6000 Nano chip assay) and a Qubit 3.0 (Quant-It dsRNA BR Assay).

Total RNA was rRNA-depleted using the Ribo-Zero rRNA Removal Kit (Gram Negative Bacteria) (Illumina) and cDNA libraries were obtained using the TruSeq RNA kit following Illumina’s recommendations. Briefly, rRNA-depleted RNA was chemically fragmented prior to reverse transcription and cDNA generation. The cDNA fragments then went through an end repair process, the addition of a single “A” base to the 3′ end and then ligation of the adapters. Finally, the products were purified and enriched by PCR to create the indexed final double stranded cDNA library. The pool of libraries was sequenced on an Illumina HiSeq 2500 sequencer. The quality control of the raw reads was performed using FastQC^2^ program as previously described (Matanza and Osorio, 2018). The raw pair-end reads were mapped against the reference genome of the *Pdd* type strain CIP102761 (GenBank accession number NZ_ADBS00000000.1), using Bowtie2 (Langmead and Salzberg, 2012) v2.2.6 algorithm. Several quality control steps were performed. Reads displaying a very low quality were removed by using Samtools (Li et al., 2009) and Picard Tools software^3^. Furthermore, one of the key factors that can condition the sequencing process is the GC content of samples which was checked as normal (distribution between 40 and 60%) in our experiment. Likewise, distribution of duplicates was evaluated to confirm the normal small proportion. The process of genetic quantification was carried out by the HTSeq (Anders et al., 2015) software (0.6.1 version).

Concordance between samples of the same condition (replicates of each of the two assayed temperatures) was carried out by a study of correlation and distance considering the whole transcriptome normalized by the size of the library. This process was made using the statistics program R. Differential expression analysis was assessed using DESeq2 (Love et al., 2014) method (1.18.1 version). The analysis of Differentially Expressed Genes (DEG) was done by using statistical packages designed by Python and R, using the DESseq2 (Love et al., 2014) algorithm applying a differential negative binomial distribution for the statistics significance. Comparison between the two different conditions (37°C vs. 25°C) was set as fixed effect in DEseq2. A Python script developed at Sistemas Genómicos (Valencia, Spain) was employed to generate a data matrix for each group condition with the counts obtained from HTSeq count for each sample (each of the three replicates at each of the two temperatures). Genes with Fold Change (FC) value lower than -1.5 or higher than 1.5 and a *P*-value adjusted by False Discovery Rate (FDR) ≤ 0.05 were considered as differentially expressed. FPKM (Fragments per kilobase per million fragments mapped) values calculated with Cufflinks v2.2.1 (Langmead and Salzberg, 2012) were used to calculate the expression of each individual gene. FPKM is used for normalization of the data since it indicates the number of lectures of a given gene per kilobase (independently of the length of the gene), and per million reads (independently of the size of the library).

### Construction of Insertional Mutants in Heat-Shock Related Genes

For insertional mutation of *clpB*, *groEl*, and *htpG* genes encoding heat-shock proteins, an internal fragment of each gene was PCR amplified and cloned into the suicide vector pNidKan (Mouriño et al., 2004). The mutant allele constructions were mobilized from *Escherichia coli* S17-1-λpir into the *Pdd* parental strain RM-71. Insertions of the suicide vectors into the chromosome by a single crossover would result in a Km^r^ phenotype and disruption of the coding sequence. The insertional mutants were selected on thiosulfate-citrate-bile-sucrose (TCBS) agar plates containing kanamycin (50 mg L^–1^), and the presence of the plasmid inserted into the target gene was confirmed by PCR. Mutants were grown in parallel with the parental strain RM-71 at 25 and 37°C to assess differences in growth, using the spectrophotometer Epoch2 microplate reader (Biotek).

### Screening of a Mini-Tn*10* Transposon Insertional Library

A mini-Tn*10* transposon mutant library of approximately 2,000 clones was generated in *Pdd* parental strain RM-71 in a previous study (Terceti et al., 2017), and clones were always grown at a temperature of 25°C. The library, which consisted of clones maintained frozen at -80°C in 96-well plates, was used in the present study for the screening of putative mutant clones exhibiting impaired growth at 37°C. To this aim, each 96-well plate was carefully thawed, and clones were replicated into 96-well plates containing fresh TSB-1 medium. Two replicas per plate were incubated at 25 and 37°C respectively, and bacterial growth was monitored for 24 h using the spectrophotometer Epoch2 microplate reader (Biotek).

### β-Galactosidase Assays

RM-71 strain derivatives carrying the *dly*, *hlyA*_*pl*_, and *hlyA*_*ch*_ hemolysin gene promoters fused to a promotorless *lacZ* gene in reporter plasmid pHRP309 were grown in TSB-1 at 25 and 37°C, and β-galactosidase activities measured as previously described (Rivas et al., 2013). Three independent experiments with 3 replicates each were conducted. The statistical analysis of the expression data was carried out with the Student’s *t*-test (adjusted *P* < 0.05). Mann–Whitney test was used for non-parametric comparison of the mean values.

## Results

### Growth at 37°C Causes Impairment in Viability, Changes in Cell Morphology, Reduced Tolerance to Benzylpenicillin, and Modification of Membrane Lipid Composition

In order to cause disease in a human host, a marine bacterium has to be able to replicate at temperatures near 37°C. The ability to grow at temperatures > 30°C is indeed a differential phenotypic trait of *P. damselae* subsp. *damselae* in comparison to its sibling subspecies, the subsp. *piscicida* (*Pdp*). This difference is supposed to contribute to the ability of *Pdd* to colonize and establish an infection in homeothermic animals, whereas *Pdp* is pathogenic only for fish (Romalde, 2002).

**FIGURE 1:**
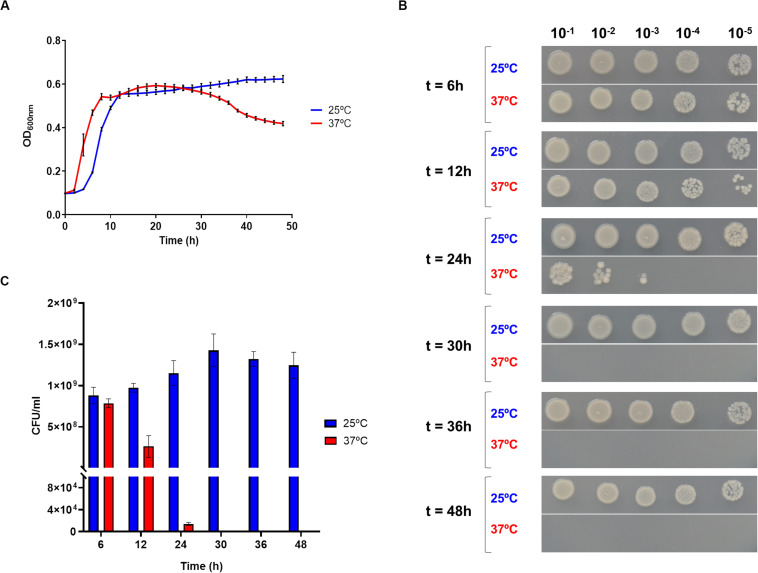
Growth at 37°C impairs cell survival in *Pdd*. **(A)** Growth curves of *Pdd* RM-71 at 25 and 37°C in TSB-1, showing the brief stationary phase and the start of the death phase at 37°C, contrasting with the prolonged stationary phase at 25°C. Mean data of three independent experiments are shown. **(B)** Growth at 37°C causes a drastic reduction in CFU numbers as detected by drop-plate assays. Serial decimal dilutions of cultures at 25 and 37°C were prepared from samples taken at 6, 12, 24, 30, 36, and 48 h incubation, and 5 μl aliquots were applied as drops onto TSA-1 plates and incubated at 25°C for 24 h. **(C)** Quantitative representation of the drop-plate assay data. Viable counts are zero after 30 h cultivation at 37°C whereas at 25°C the number of CFUs is still increasing.

**FIGURE 2:**
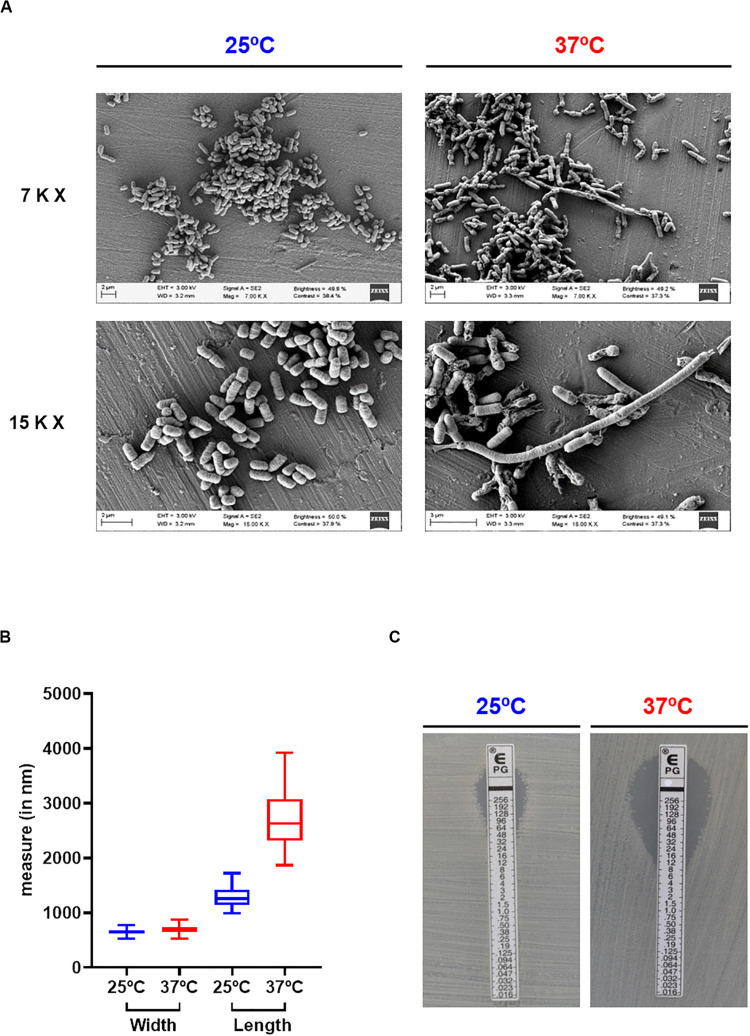
Growth at 37°C impairs cell morphology and benzylpenicillin tolerance in *Pdd*. **(A)** Scanning electron microscopy images showing the increase in cell length and the impairment in daughter cell separation in *Pdd* cells grown at 37°C with respect to the normal cell size at 25°C. Pictures from two magnifications, 7,000× (7 K X) and 15,000× (15 K X) are shown. **(B)** Box plot graphs showing cell width and cell length values at 25 and at 37°C. Whiskers indicate min and max values. Data correspond to the measurements of 50 independent cells per temperature assayed. **(C)** *E*-tests for benzylpenicillin of *Pdd* RM-71 cultivated at 25 and 37°C, showing the decreased tolerance to this antimicrobial compound at 37°C.

To assess the effect of temperature in *Pdd* growth, the highly virulent strain RM-71, isolated from a disease outbreak in a turbot farm (Fouz et al., 1992), was cultured at 25 and 37°C for 48 h. Cultivation at 37°C triggered an earlier entry into exponential phase than at 25°C, but OD_600_ values dropped after 24 h suggesting a cell death scenario (Figure 1A). In order to test this hypothesis, viable cells were quantified by drop-counting on agar plates. As a result, there was a > 3.5-fold reduction in the number of colony-forming units (CFU) at 12 h incubation in cultures at 37°C in comparison to 25°C. Notably, CFU were no longer detected from cultures at 37°C after 30 h (Figures 1B,C). The observation of cell viability reduction at 37°C prompted us to study cell morphology by scanning electron microscopy, in exponentially-growing (OD_600_ of 0.55) cultures at 25 and 37°C. Remarkably, cells grown at 25°C showed normal rod morphology by scanning electron microscopy analysis whereas cells grown at 37°C exhibited chain-like structures and aberrant long shapes suggesting a defect in daughter cell separation and in septum formation (Figure 2A). Quantitatively, mean cell length values at 37°C were significantly higher than at 25°C (Figure 2B). In addition, growth at 37°C caused a reduction in the intrinsic tolerance of *Pdd* RM-71 to benzylpenicillin in comparison to 25°C (Figure 2C).

Bacteria can adapt to variations in environmental temperature by altering the ratio between saturated (SFA) and unsaturated (UFA) fatty acids in membranes (Marr and Ingraham, 1962). However, the effects of temperature in membrane lipids composition in *Pdd* have not been studied so far. We here carried out an analysis of fatty acid methyl esters (FAME) from *Pdd* RM-71 cultures at 25 and 37°C. We observed that the percentage of saturated fatty acids in membranes increased at 37°C with respect to 25°C, and the three saturated fatty acids 16:0 N alcohol, 16:0 iso and 19:0 cyclo w8c were exclusively detected at 37°C (Supplementary Table S1).

### Human Isolates Are Not Better Adapted for Growth at 37°C Than Fish Isolates, and Comparative Genomics Does Not Reveal Human-Specific Gene Markers

**FIGURE 3:**
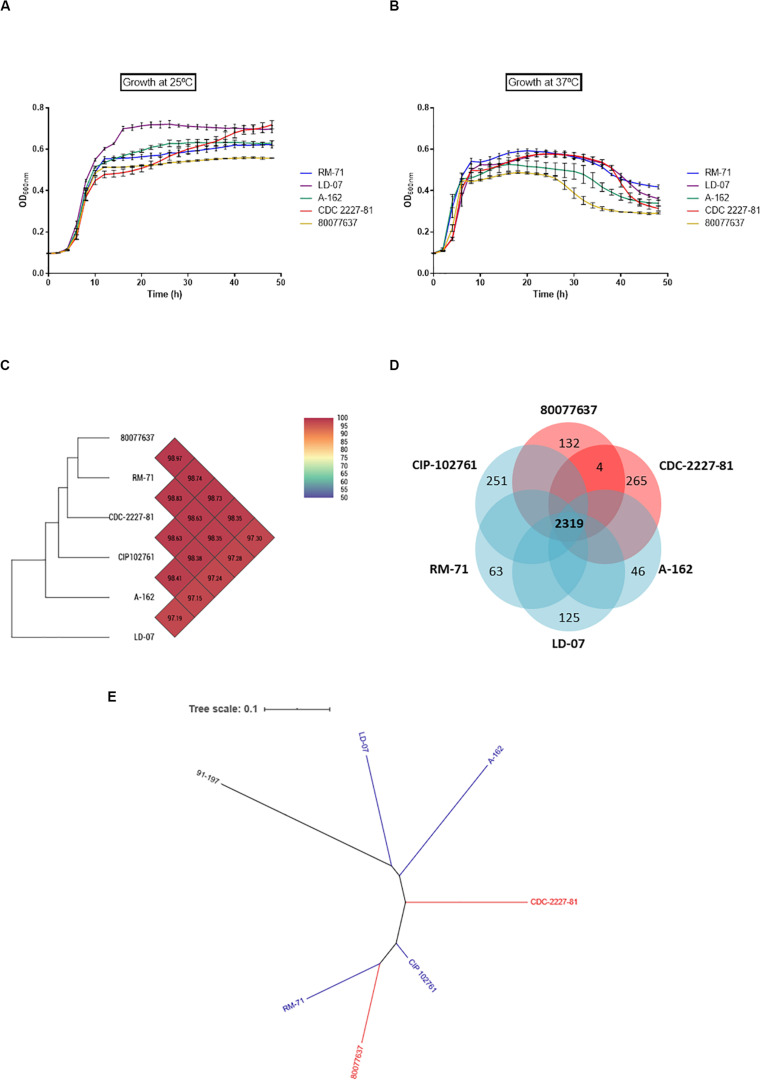
*Pdd* isolates from human origin do not exhibit an advantage for growth at 37°C compared to fish isolates, and do not contain specific gene markers unique to human isolates. Growth curves of *Pdd* strains from human (CDC 2227-81; 80077637) and fish (RM-71; LD-07; A-162) origin, cultivated at 25°C **(A)** and at 37°C **(B)**. Note that human clinical strain 80077637 achieves the least OD_600_ values of all strains at the two temperatures. **(C)** Heatmap generated by comparison of core genes with OrthoANI values calculated from the OAT software shows the absence of close phylogenetic association of human isolates vs. fish isolates. **(D)** Venn diagram depicting the comparative genomics of six *Pdd* strains. The core genome was estimated in 2319 genes, and each isolate has a varying number of strain-unique genes, ranging from 46 unique genes to strain A-162 to the 265 unique genes to strain CDC-2227-81. Of note, the two human-isolated strains only share 4 specific genes which are absent from the fish strains. **(E)** Dendrogram of *Pdd* strains based on genomic BLAST, showing that human clinical strains do not form an independent clade with respect to fish isolates. The genome of the sibling subspecies *P. damselae* subsp. *piscicida* strain 91–197 was included for comparative purposes. The genomic BLAST file was downloaded from the NCBI database and the tree was visualized by Interactive Tree Of Life (iTOL v5). The genome of the *Pdd* type strain CIP 102761 was included in the genomic analyses depicted in **(C–E)**.

We selected two strains from human clinical cases (CDC 2227-81 and 80077637), and three strains from diseased fish (RM-71, LD-07, and A-162), and growth of all the isolates was monitored at 25 and 37°C. The ability to grow at 37°C was not a differential trait of human isolates since all the strains showed similar dynamics: early exponential growth and a drop in the OD_600_ after approximately 24 h (Figures 3A,B). Of note, human strain 80077637 achieved the least OD_600_ values at the two temperatures. These data, together with the above described survival assays, suggest that 37°C, rather than being a normal condition, represents a stress condition for *Pdd*.

To assess the existence of gene markers characteristic of human isolates, we here obtained for the first time the draft genome sequences of two *Pdd* strains isolated from humans, CDC-2227-81 (Kreger, 1984) and 80077637 (Hundenborn et al., 2013), and a comparative genomics analysis was conducted including the genomes of four fish isolated strains, RM-71, LD-07, A-162, (Vences et al., 2017) and CIP102761. Ortho Average nucleotide identity (OrthoANI) values, which are derived from the pairwise comparison of strains taking into consideration the core genes shared by all the strains, ranged from 97.19 to 98.97 between strains (Figure 3C). Two genomes are considered the same species when the ANI value is higher than 95–96% (Lee et al., 2016). The 4 strains harboring the virulence plasmid pPHDD1 clustered together independently of their source of isolation, and human isolates were not more similar between them than to the other genomes, when core genes where compared. In fact, the highest similarity value was found between RM-71 (fish isolate) and 80077637 (human isolate). Notably, genomics analysis unveiled a large number of strain-specific genes, with 265 genes unique to strain CDC-2227-81 and 132 genes unique to strain 80077637 (Figure 3D). Four genes were common to the human isolates and absent in the fish isolates. These genes encoded a racemase (locus F6450_06750), an HD domain-containing protein (F6450_11305), a MFS transporter (F6450_16290), and a multidrug transporter (F6450_07685) (GenBank loci tags correspond to CDC 2227-81). However, BLAST searches in GenBank database unveiled that three of these genes were also present in other *Pdd* strains isolated from fish, and the multidrug transporter F6450_07685 was found in a *Pdd* isolate from the porpoise *Neophocaena asiaeorientalis* and in non-pathogenic *Photobacterium* species (data not shown). A phylogenetic tree constructed based on the genome alignments (including core and accessory genome) of *Pdd* strains revealed that human-isolated strains do not represent an independent evolutionary line with respect to the fish isolates, and human clinical strain 80077637 fell into the same clade with fish-isolated RM-71 (Figure 3E).

### RNA Sequencing Reveals Major Changes in the *Pdd* Transcriptome in Response to Growth at 37°C

**FIGURE 4:**
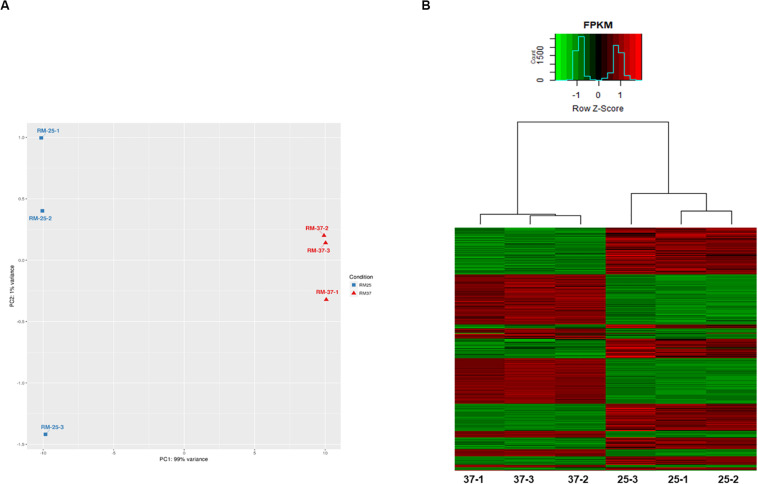
RNA sequencing reveals changes in the *Pdd* transcriptome in response to growth at 37°C vs. 25°C. **(A)** Hierarchical clustering of all genes by Principal Component Analysis (PCA) demonstrates clear separation of the 2 sample groups (25°C vs. 37°C). **(B)** Heat map with gene expression plotted as z-score normalized FPKM (Fragments Per Kilobase of transcript per Million mapped reads) value for differentially expressed genes (DEGs). The hierarchical clustering shows the different expression profiles of RM-71 at the two temperatures and provides strong evidence of the grouping of the biological replicates.

The RNAseq analysis of RM-71 resulted in 1607 differentially expressed genes (DEGs): 804 upregulated (Foldchange (FC) higher than 1.5) and 803 downregulated (FC lower than −1.5) at 37°C with respect to 25°C (Supplementary Table S2). The principal component analysis (PCA) showed that samples grown at each different temperature were distinguishable and replicates clustered together (Figure 4A). A heat map with gene expression plotted as z-score normalized FPKM (Fragments Per Kilobase of transcript per Million mapped reads) value for differentially expressed genes (DEGs) was performed. Hierarchical clustering showed the different expression profiles of RM-71 at the two temperatures and the grouping of the biological replicates (Figure 4B).

### Growth at 37°C Triggers a Strong Stress Response: Heat Shock Proteins and Defense-Related Mechanisms Are Upregulated at 37°C

**FIGURE 5:**
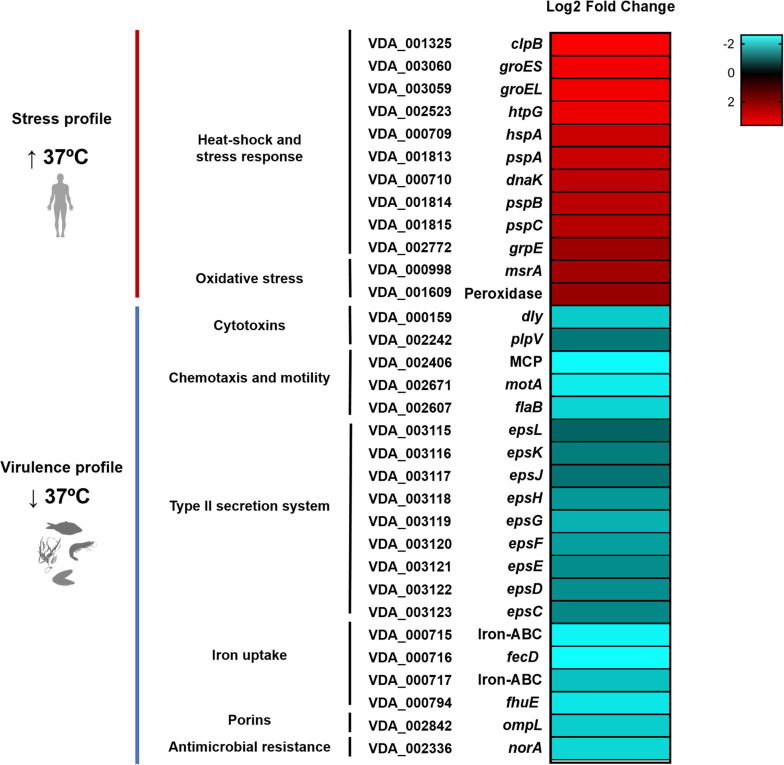
Heatmap of 32 selected DEGs of the *Pdd* transcriptome, including the main upregulated genes at 37°C in the categories of heat-shock and stress response mechanisms, and the main downregulated genes related to virulence properties. Cultivation of *Pdd* at 37°C triggers a strong stress response, indicating that human body temperature represents a stressful condition for this marine pathogen. Conversely, growth at 37°C downregulates the virulence gene expression profile with respect to 25°C, corroborating that 25°C is the optimal temperature for the pathogenic lifestyle of *Pdd* in the marine environment. VDA gene tags correspond to the complete genome sequence of the *Pdd* type strain CIP 102761.

To better illustrate the impact of human body temperature on gene expression, a heat map was generated with 32 selected DEGs that showed remarkable FC values (Figure 5). Notably, heat-shock proteins and molecular chaperones were among the most upregulated genes at 37°C (Table 1 and Figure 5). The genes encoding ClpB, GroEl and HtpG heat-shock proteins were selected for construction of insertional mutants, using the suicide plasmid pNidKan (Mouriño et al., 2004) containing an internal fragment of the target gene. A *htpG* insertional mutant was successfully generated although it did not show any impairment in growth at 37°C compared to 25°C (data not shown). Notably, we were unable to obtain insertional mutants for *clpB* and *groEL* despite numerous attempts, suggesting that these genes are essential, even for growth at 25°C (the temperature at which strains were incubated during the mutant construction process). We thus aimed to gain a further insight into the existence of gene markers essential for growth at 37°C but dispensable at 25°C. Identification of such markers would be of interest for the design of control strategies of human infections caused by *Pdd*. For this, we screened a > 2000-clone Tn*10* transposon mutant library of *Pdd* RM-71 constructed in a previous study (Terceti et al., 2017), and each clone was grown in TSB-1 in 96-well plates per duplicate at 25 and at 37°C. However, all the mutant clones were able to grow at 37°C without any evident signs of impairment (data not shown), which suggests that any potential transposon-insertional mutation that would prevent growth at 37°C, would also be deleterious for growth at 25°C (the temperature at which the transposon library was originally obtained).

**TABLE 1:** List of selected top DEGs whose expression is upregulated at 37°C.

Gene ID	Product/Function	Fold change	*p*-value	Location
**Stress response and defense mechanisms**				
VDA_001325	ClpB protein	12.1	0	ChrI
VDA_003060	Heat shock protein 60 family co-chaperone GroES	10.4	0	ChrI
VDA_003059	Heat shock protein 60 family chaperone GroEL	10.4	0	ChrI
VDA_002523	Chaperone protein HtpG	10.1	0	ChrI
VDA_000709	Heat shock protein A	7.3	0	ChrII
VDA_001813	Phage shock protein A	7.1	8.97261E−76	ChrI
VDA_000710	Chaperone protein DnaK	6.4	0	ChrII
VDA_001814	Phage shock protein B	6.1	1.624E−197	ChrI
VDA_001815	Hypothetical phage shock protein C	5.9	1.7736E−117	ChrI
VDA_002771	Chaperone protein DnaK	5.9	0	ChrI
VDA_003125	Heat-shock chaperonin	5.5	0	ChrI
VDA_000998	Peptide methionine sulfoxide reductase MsrA	4.9	0	ChrII
VDA_001609	Alkyl hydroperoxide reductase subunit C-like protein	4.4	0	ChrI
VDA_002772	Heat shock protein GrpE	4.6	0	ChrI
VDA_000104	Lipoprotein precursor OmpA family	5.4	5.73249E−76	pPHDD1
**Metabolism**				
VDA_002723	Glyoxylate cycle isocitrate lyase	4.0	1.1169E−114	ChrI
VDA_001102	ATP synthase gamma chain	4.1	8.8225E−67	ChrII
VDA_001103	ATP synthase alpha chain	3.5	1.55568E−69	ChrII
VDA_002810	Phosphopentomutase	3.2	1.7648E−261	ChrI
VDA_002812	Deoxyribose-phosphate aldolase	4.8	0	ChrI
VDA_002811	Thymidine phosphorylase	3.6	7.7733E−277	ChrI
VDA_003271	Thiamine biosynthesis ThiC	4.5	1.325E−176	ChrI
VDA_002967	Thiamine ABC transporter	4.4	1.3878E−296	ChrI
VDA_002261	NAD-dependent glyceraldehyde-3-phosphate dehydrogenase	13.5	0	ChrI
VDA_003183	Oligopeptidase A	5.5	0	ChrI
VDA_002812	Deoxyribose-phosphate aldolase	4.8	0	ChrI
VDA_000814	Hypothetical protein (DUF 4832)	12.5	1.2613E−200	ChrII
VDA_002964	3-isopropylmalate dehydratase large subunit	7.1	0	ChrI
VDA_002965	3-isopropylmalate dehydratase small subunit	7.1	0	ChrI
VDA_002963	3-isopropylmalate dehydrogenase	6.6	0	ChrI
VDA_001041	Adenylosuccinate synthetase	6.5	1.6314E−128	ChrII
VDA_002962	2-isopropylmalate synthase	6.3	0	ChrI
VDA_002881	Homoserine kinase	4.7	0	ChrI
VDA_002880	Threonine synthase	4.7	0	ChrI
**Transporters, adaptation, and colonization**				
VDA_002633	Sulfate transporter CysZ	3.1	3.0492E−158	ChrI
VDA_003428	Gluthatione-regulated potassium-efflux system protein KefB	3.4	4.4457E−183	ChrI
VDA_000372	Putative choline-glycine betaine transporter	7.7	0	ChrII
VDA_002854	Na^(+)^/H^(+)^ antiporter NhaA type	4.7	6.0472E−303	ChrI
VDA_000302	Urease accessory protein UreG	3.2	2.30662E−44	ChrII
VDA_000303	Urease accessory protein UreF	4.8	2.62317E−51	ChrII
VDA_000304	Urease accessory protein UreE	4.8	2.62317E−51	ChrII
VDA_000305	Urease subunit alpha UreC	3.5	4.26325E−17	ChrII
VDA_000306	Urease gamma subunit UreA	3.3	1.4581E−58	ChrII
**Lipases**				
VDA_000412	Hypothetical protein (patatin similar to Yjju protein)	10.6	0	ChrII
VDA_002140	Putative lipase/esterase protein	3.5	2.11351E−81	ChrI
**Pilus assembly**				
VDA_000103	Protein TadG associated with Flp pilus assembly	5.4	1.55701E−93	pPHDD1
VDA_000134	IncF plasmid conjugative transfer pilus assembly protein TraF	5.0	3.42729E−12	pPHDD1
VDA_000102	Flp pilus assembly protein TadD	4.9	1.09531E−19	pPHDD1
VDA_000142	Mfp1 (mar binding filament-like protein 1)	4.9	0	pPHDD1
**Transcriptional regulators and DNA-binding proteins**				
VDA_001014	Hypothetical transcriptional regulator	5.7	6.7055E−294	ChrII
VDA_000143	DNA-binding protein H-NS	5.0	0	pPHDD1
VDA_001042	Transcriptional regulator LysR family	4.6	3.1515E−180	ChrII
VDA_000105	Site-specific recombinase resolvase family	8.3	1.925E−296	pPHDD1
VDA_000152	Chromosome segregation ATPase	5.0	9.68836E−64	pPHDD1

*Enhanced expression at 37°C is denoted by positive Fold change (FC) values. Genomic location of each DEG in either ChrI (chromosome I), ChrII (chromosome II) or in virulence plasmid pPHDD1 is detailed.*

Genes involved in prevention of oxidative damage were found among the most up-regulated at 37°C (Table 1 and Figure 5), including the peptide methionine sulfoxide reductase MsrA, alkyl hydroperoxide reductase subunit C-like protein and the thioredoxin peroxidase, among others. These enzymes are produced by bacteria as defensive strategies against host defenses. Together with the viability assays, this association between high temperatures and upregulation of stress and defense mechanisms supports the idea that 37°C constitutes a stressful condition for *Pdd*.

### Metabolic Pathways, Transporters, ATP Generation, and Nutrient Acquisition Are Upregulated at 37°C

Cultivation at 37°C causes a rapid initial growth of *Pdd* as shown above. In the RNA seq analysis, the expression of genes encoding enzymes of catabolic and anabolic pathways were strongly up-regulated at 37°C (Table 1), being the glycolytic enzyme NAD-dependent glyceraldehyde-3-phosphate dehydrogenase one of the most strongly upregulated genes at 37°C. Leucine, isoleucine, threonine and thiamine biosynthetic genes, enzymes of the phosphate pentose pathway and the purine/pyrimidine metabolism were upregulated at 37°C, likely to supply precursors for proteins, nucleotides and coenzymes to sustain the rapid initial growth of *Pdd* observed at 37°C. Accordingly, genes encoding the ATP-synthase subunits were upregulated. A number of pH-dependent and independent transporters were among the most upregulated genes at 37°C, including a Na^+^/H^+^ antiporter of NhaA-type. In *V. cholerae* this gene contributes to resistance to Na^+^ at alkaline pH leading to improved fitness in the environment (Herz et al., 2003). Also upregulated were several sulfate transporters, and the potassium-efflux system protein KefB. Of relevance, *kefB* belongs to the locus of heat resistance in *E. coli* (Mercer et al., 2017) and it has been studied for its ability to acidify intracellular pH in response to electrophilic stress (Ferguson et al., 1995).

*Pdd* is one of the few urease-producer species within the *Vibrionaceae* family, and genes encoding urease subunits were upregulated at human body temperature. Urease is a well-studied enzyme in gastric pathogens for its role in maintaining periplasmic pH near neutrality in highly acidic environments (Sachs et al., 2003) and it also constitutes a key virulence factor of numerous urinary pathogens (Nielubowicz and Mobley, 2010; de Paiva-Santos et al., 2018). To date no functional studies of *ure* genes have been carried out in *Pdd*. This up-regulation of *ure* genes might play a role in some *Pdd* infections as this pathogen has been isolated as causative agent of a urinary tract infection in a pregnant woman in warm coastal areas of the Caribbean Sea (Alvarez et al., 2006).

Genes encoding degradative functions were upregulated, including several putative lipases (VDA_000412 and VDA_002140) and an operon for utilization and transport of galactosamine and glycosaminoglycans (VDA_001072-VDA_001083). Glycosaminoglycans are major components in extracellular matrix of animals (Gandhi and Mancera, 2008). *Pdd* is considered a generalist pathogen of a wide range of animals (Osorio et al., 2018) so genetic networks involved in the utilization of different carbon sources allows colonization of different niches and hosts. At 37°C, *Pdd* also upregulated genes of two pili structures encoded within the virulence plasmid pPHDD1, including the *tra* genes for conjugative pilus biogenesis, and the genes of the Flp/Tad pilus, with a potential role in adhesion/colonization.

### A Virulence Plasmid-Encoded H-NS Repressor Is Upregulated at 37°C

How *Pdd* modulates genetic expression in response to external conditions remains unknown. Several LysR family transcriptional regulators were upregulated at 37°C (VDA_001014, VDA_001042, VDA_000547, and VDA_001035). Of special relevance is the observation that the DNA-binding protein H-NS (VDA_000143), encoded within the virulence plasmid pPHDD1 showed a > fivefold upregulation at 37°C. In *Vibrio cholerae*, this protein functions as a virulence repressor controlling motility and biofilm formation (Wang et al., 2015). As detailed below, growth at 37°C was found to downregulate virulence and motility functions in *Pdd*.

### Growth at 37°C Causes Downregulation of Virulence Factors

**FIGURE 6:**
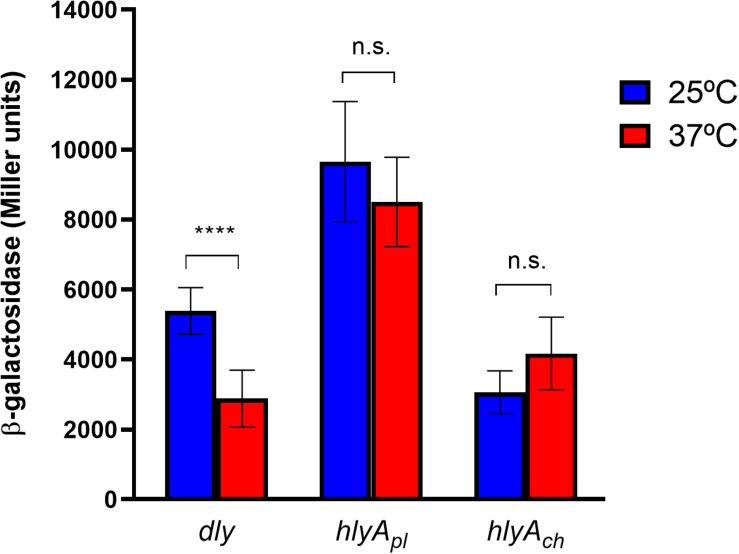
Transcription activities of the three major cytotoxin genes of *Pdd*, damselysin (*dly* gene), phobalysin P (*hlyA*_*pl*_ gene), and phobalysin C (*hlyA*_*ch*_ gene) under the temperature conditions of a human host (37°C) in comparison to the temperature in warm seawaters (25°C), as determined by transcriptional fusions of *dly, hlyA*_*pl*_, and *hlyA*_*ch*_ promoters to *lacZ* reporter gene. The β-galactosidase values (expressed in Miller units) show that *dly* is significantly downregulated at 37°C whereas expression of *hlyA*_*pl*_ and *hlyA*_*ch*_ is not significantly affected at 37°C vs. 25°C, thus validating the results of the transcriptomics analysis. ****denotes the degree of significance in the Mann-Whitney test (*p* value was <0.0001).

In a previous work, we demonstrated that many *Pdd* virulence factors were up-regulated at 25°C in comparison with 15°C, thus contributing to partially explain the onset of disease outbreaks in marine fish farms when seawater temperature is close to 25°C (Matanza and Osorio, 2018). We here investigated how human body temperature affects virulence gene expression in *Pdd*. Of note, well-known virulence factors of *Pdd* were downregulated at 37°C, suggesting that infection of a human host is not a condition that has been favored during evolution of this marine pathogen (Table 2 and Figure 5). Damselysin (Dly) toxin, a major virulence factor of *Pdd*, showed a fourfold down-regulation at 37°C. The two pore-forming toxins PhlyP and PhlyC did not show a differential expression at 37°C, and the phospholipase PlpV showed downregulation at 37°C. β-galactosidase assays of transcriptional fusions of the hemolysin gene promoters to *lacZ* gene validated the RNA-seq results (Figure 6). Despite the absence of upregulation at 37°C, the three major cytotoxins Dly, PhlyP, and PhlyC exhibited high FPKM values (Supplementary Table S3), in accordance with previous studies that demonstrated that the three cytotoxin genes are highly expressed and the toxins represent a major fraction of the *Pdd* secretome (Matanza and Osorio, 2018; Terceti et al., 2019). Genes of the type II secretion system (*eps* genes, VDA_003115-VDA_003123) that play a major role in the secretion of the cytotoxins and therefore in virulence (Rivas et al., 2015a; Vences et al., 2017), were downregulated at 37°C (Figure 5). The two-component RstAB system, a major positive regulator of virulence in *Pdd* (Terceti et al., 2017, 2019), was not significantly affected by temperature.

**TABLE 2:** List of selected top DEGs whose expression is downregulated at 37°C.

Gene ID	Product/Function	Fold change	*p*-value	Location
**Defense and virulence**				
VDA_000717	Iron ABC-transporter	–3.9	2.7907E−177	ChrII
VDA_000716	Iron(III) dicitrate transport system permease protein FecD	–6.1	6.4554E−244	ChrII
VDA_000715	ABC type periplasmic iron siderophore/cobalamin binding protein	–5.6	0	ChrII
VDA_000794	Ferrichrome-iron receptor	–5.1	0	ChrII
VDA_000159	Damselysin	–4.2	8.6351E−269	pPHDD1
VDA_002336	Multidrug efflux protein NorA	–4.6	1.7265E−164	ChrI
VDA_002842	OmpL porin-like protein L precursor	–4.3	4.4961E−185	ChrI
VDA_000341	ABC transporter periplasmic spermidine putrescine-binding protein PotD	–7.9	0	ChrII
VDA_001897	Thiol-disulfide isomerase	–4.6	6.0694E−145	ChrI
VDA_002117	ABC transporter periplasmic spermidine putrescine-binding protein PotD	–4.2	7.62485E−75	ChrI
VDA_003028	Flavohemoprotein	–4.2	0	ChrI
VDA_000342	Oxidoreductase	–3.2	4.73193E−72	ChrII
**Cold response**				
VDA_003169	Cold shock protein	–16.3	0	ChrI
VDA_000346	Putative Cold shock-like protein	–8.4	3.1036E−252	ChrII
VDA_000863	Cold-shock DEAD-box protein A	–5.3	9.3703E−299	ChrII
**Hypothetical proteins of unknown function**				
VDA_002460	Lipoprotein putative	–15.9	0	ChrI
VDA_002316	Hypothetical protein (Helix-turn-helix domains)	–14.2	0	ChrI
VDA_001898	Putative heat shock protein YegD	–7.3	7.2519E−209	ChrI
**Histone acetylation**				
VDA_000822	Histone acetyltransferase HPA2	–10.8	4.0491E−204	ChrII
**Flagellar motility and chemotaxis**				
VDA_002406	N-acetylglucosamine regulated methyl-accepting chemotaxis protein	–6.0	5.4912E−139	ChrI
VDA_002671	Flagellar motor rotation protein MotA	–5.4	1.0403E−179	ChrI
VDA_001613	Sodium-type flagellar protein MotY precursor	–4.9	1.5077E−180	ChrI
VDA_003029	Sodium-type polar flagellar protein MotX	–4.8	3.9208E−150	ChrI
VDA_001198	Putative methyl-accepting chemotaxis protein	–4.1	1.6095E−186	ChrI
VDA_003044	Methyl-accepting chemotaxis protein	–4.0	3.2241E−137	ChrI
VDA_002619	Chemotaxis protein CheV	–3.4	6.3682E−259	ChrI
VDA_002670	Flagellar motor rotation protein MotB	–4.1	9.0774E−129	ChrI
VDA_002607	Flagellin protein FlaB	–4.5	2.4193E−132	ChrI
**Porins, permeases, and transporters**				
VDA_001789	Nucleoside permease NupC	–10.7	0	ChrI
VDA_001677	Sulfate permease	–6.5	0	ChrI
VDA_002944	Probable low-affinity inorganic phosphate transporter	–6.8	0	ChrI
VDA_002943	Phosphate transport regulator	–7.4	0	ChrI
**Transcriptional regulators**				
VDA_003227	Putative transcriptional regulator DeoR family protein	–3.4	1.2526E−136	ChrI
VDA_001543	Hypothetical response regulator	–3.7	2.486E−250	ChrI
VDA_002957	Putative LuxZ	–4.6	0	ChrI
**Metabolism**				
VDA_002716	Myo-inositol-1(or 4)-monophosphatase	–7.4	1.66767E−53	ChrI
VDA_001806	Orotidine 5′-phosphate decarboxylase	–5.1	2.4264E−246	ChrI
VDA_003379	Fructose-1.6-bisphosphatase GlpX type	–4.5	4.51805E−64	ChrI
VDA_001717	Cytochrome c-type protein TorC	–4.3	3.762E−125	ChrI
VDA_000979	HNH nuclease	–10.9	3.2236E−205	ChrII
VDA_002963	Inosine-5′-monophosphate dehydrogenase	–6.6	0	ChrI
VDA_003340	Orotate phosphoribosyltransferase	–4.1	1.4272E−176	ChrI
**Adjustment of membrane composition**				
VDA_002169	Phosphatidylglycerophosphatase B	–3.9	1.8426E−114	ChrI
VDA_002932	1-acyl-sn-glycerol-3-phosphate acyltransferase	–3.5	3.0484E−200	ChrI
VDA_002087	3-hydroxydecanoyl-[acyl-carrier-protein] dehydratase	–4.2	0	ChrI
VDA_002463	UDP-2.3-diacylglucosamine hydrolase	–4.1	6.7473E−233	ChrI

*Note that downregulated expression at 37°C is denoted by negative fold change values.*

Other virulence-related functions were downregulated at 37°C (Table 2 and Figure 5): an iron ABC-transporter (VDA_00715-VDA_000717), a TonB-dependent siderophore receptor (VDA_000794), and the *potABCD* operon (VDA_002114-VDA_002117, VDA_000341) encoding a polyamine (spermidine and putrescine) transport system. Polyamines are essential for normal growth and proliferation in bacteria (Shah and Swiatlo, 2008). In *Vibrio vulnificus* expression of *pot* genes is up-regulated in presence of human serum (Williams et al., 2014) and in *V. cholerae* they regulate biofilm formation (McGinnis et al., 2009).

Porins are outer membrane proteins that modulate pathogenicity of many *Vibrio* species. VDA_002842 is downregulated at 37°C and shows similarity to *Vibrionaceae* porins, including OmpU. OmpU has been pointed for its role in adhesion, host recognition and pathogenesis in several *Vibrio* species (Goo et al., 2006; Duperthuy et al., 2011; Sakharwade and Mukhopadhaya, 2015). Multidrug efflux protein VDA_002336 showed a down-regulation at 37°C. It belongs to the multidrug and toxic-compound extrusion (MATE) proteins group, involved in protecting cells against antibiotics and other substances in human pathogenic *Vibrios* as *Vibrio parahaemolyticus* and *Vibrio cholerae* (Begum et al., 2005; Otsuka et al., 2005).

Motility and adhesion to hosts are considered of special relevance in *Pdd* pathogenicity (Fouz et al., 2000; Rivas et al., 2015b). A recent study demonstrated that disruption of chemotaxis regulator *cheA* reduced motility and production of PhlyP, and subsequently impaired bacterial adhesion to human cell lines (von Hoven et al., 2018). The current study revealed a large set of genes with a role in chemotaxis and flagellar motility which were downregulated at 37°C (Table 2 and Figure 5). All these data indicate that exposure of *Pdd* at human body temperature triggers a transcriptomic pattern of defense against stress rather than a high virulence profile.

### Genes Encoding Cold Shock Response, Membrane Transporters, Metabolic Functions, and Adjustment of Membrane Composition Are Downregulated at 37°C

There was a downregulation of cold shock functions at 37°C (Table 2), including two proteins that might play a role in cold tolerance in *Pdd*: VDA_002460 and VDA_002316. Phosphate, nucleoside and vitamin transporters, and permeases for inorganic ions were downregulated at 37°C (Table 2). DNA can be utilized as a source of phosphate, carbon and nitrogen. Phosphate limitation is encountered by marine pathogens in either the environment or inside the host. We have found that a phosphate transport regulator (VDA_002943) and a low-affinity inorganic phosphate transporter (VDA_002944), which shares homology with *E. coli pitA* (identity 33%), are down-regulated at 37°C. As well, nucleoside permeases NupC (VDA_001789 and VDA_002313) were also down-regulated at 37°C. A previous study (Matanza and Osorio, 2018) showed that NupC VDA_001789 presented a high up-regulation at 25°C compared to 15°C. These results suggest that *Pdd* takes advantage of the utilization of DNA as a source of biomolecules especially at 25°C (temperature at which outbreaks in fish farms are favored). An operon of type ECF (energy coupling factor) ABC transporters (VDA_002275-VDA_002277) was down-regulated at 37°C, as well as the putative vitamin transporter VDA_003148. The myo-inositol-1 (or 4) – monophosphatase (VDA_002716), which participates in myo-inositol biosynthesis, showed a remarkable down-regulation at 37°C. This function has been associated with a role in correct rRNA biosynthesis (Singh et al., 2016).

During anaerobic respiration, bacteria use alternative electron acceptors such as trimethylamine N-oxide (TMAO) to oxidize organic compounds. The cytochrome c-type protein TorC (VDA_001717) that takes part in this electronic transfer (Gon et al., 2001) shows a down-regulation at 37°C. The association between anaerobic growth and temperature in *Pdd* has not been explored to date. Functions related to nucleotide and pyrimidine biosynthesis, and of carbohydrate metabolism were also downregulated. Functions for the maintenance of lipid membranes, phospholipid biosynthesis, fatty acid biosynthesis and lipid IVA biosynthesis were markedly downregulated at 37°C. These observations might correlate with the observed changes in membrane lipid composition in *Pdd* cultured at 37°C vs. 25°C as reported above (Supplementary Table S1).

## Discussion

Different to other human pathogenic Vibrios (as *V. cholerae* and *V. parahaemolyticus*) that cause diarrhea in humans and therefore gain exit from the host, infection of a human by *Pdd* can be seen as an end-point for the bacterium. Most clinical cases evolve as wound infections that may or may not complicate into fatal necrotizing fasciitis, and there is no evident exit strategy for the successful *Pdd* genotypes to go back to the marine environment. The lines of evidence presented in this study clearly suggest that the ability of *Pdd* to infect humans is more an anecdotic circumstance than an evolutionarily selected trait. *Pdd* cells growing at human body temperature undergo stressful conditions that impair viability, control of cell shape, and size. This evidence is in agreement with earlier studies that reported the difficulties to isolate *Pdd* from bullae fluid and blood in a human clinical case, whereas streaking the specimens on blood agar plates caused hemolysis in absence of growth (Clarridge and Zighelboim-Daum, 1985). In a rapidly fatal infection case, wound specimens yielded *Pdd* in pure culture but blood cultures were negative, and it was suggested that a systemic toxin released by *Pdd* contributed to the rapid fatal outcome and that septicemia did not (Fraser et al., 1997). Other studies were unable to detect *Pdd* cells upon Gram staining of specimens from tissues in infected individuals (Coffey et al., 1986; Goodell et al., 2004). All these observations can be explained, on the one hand, by the low viability of *Pdd* at 37°C as demonstrated in the present study and, on the other hand, by the high amounts of hemolysins produced, that would accumulate in host fluids during the initial phases of infection. Accumulated hemolysins might cause further tissue damage in absence of bacterial replication. Recent studies have demonstrated that under laboratory growth conditions at 25°C, the hemolysin genes are among the most highly expressed genes in *Pdd* (Matanza and Osorio, 2018; Terceti et al., 2019), and the FPKM values obtained in the present study (Supplementary Table S3) also confirmed that the three major hemolysins, Dly, PhlyP, and PhlyC exhibit high transcription levels at 37°C. Our findings on affected cell morphology and size in *Pdd* upon growth at 37°C are in agreement with previous observations of bacilli with large dimensions in Gram-stained sections from *Pdd*-infected tissues (Coffey et al., 1986) and with aberrant cell morphologies (Clarridge and Zighelboim-Daum, 1985; Goodell et al., 2004).

Similar to other species of the genera *Vibrio* and *Photobacterium*, *Pdd* exhibits high levels of intrinsic tolerance to bactericidal inhibitors of cell wall biosynthesis, as benzylpenicillin. Recent studies reported that *Pdd* RM-71 regulatory mutants in the RstAB two-component system exhibited aberrant cell morphologies, increased cell size and length, and reduced tolerance to benzylpenicillin (Terceti et al., 2019). In the present study, growth at 37°C was also found to cause increased sensitivity to benzylpenicillin with respect to 25°C. Thus, conditions that impair cell size and shape also impair penicillin tolerance in *Pdd*. A recent study reported that tolerance to beta lactams in *Vibrio cholerae* has a pleiotropic nature, and genes involved in cell envelope biogenesis and modification also play a major role in tolerance to beta lactams (Weaver et al., 2018).

A conserved strategy used by bacteria to face variations in environmental temperature is the modulation of membrane fluidity by adjustment of lipid composition (Sinensky, 1974; Rock and Cronan, 1996; Mansilla et al., 2004). The predominant strategy consists of altering the ratio between saturated (SFA) and unsaturated (UFA) fatty acids in membranes (Marr and Ingraham, 1962; Russell, 1983; Zhang and Rock, 2008). The analysis of FAME in *Pdd* RM-71 at 25 and 37°C in the present study has demonstrated that the percentage of saturated fatty acids in membranes increases at 37°C with respect to 25°C, and three saturated fatty acids are exclusively detected at 37°C. It is conceivable that the ability of this marine pathogen to inhabit different environments may rely on its skills to reversibly modify its membranes, assuring the survival outside and inside the diversity of hosts it can successfully colonize.

On the light of the current study, it is not possible to propose gene markers specific for human-adapted strains vs. fish-adapted strains in *Pdd*. The pPHDD1 virulence plasmid shared by the two human isolates analyzed here, also occurs in fish isolates (Rivas et al., 2011). A close look at the hemolytic activities of human isolates reported in an early study (Kreger, 1984) suggests that some human isolates fall within the category of low hemolytic activity (isolates that lack pPHDD1). These lines of evidence suggest that any *Pdd* genotype thriving in the marine environment might potentially cause an opportunistic infection in humans.

Recent transcriptomic studies have revealed the connection between temperature and virulence in marine pathogenic bacteria (Williams et al., 2014; Kong et al., 2017; Huang et al., 2018). In the current work, we demonstrate that *Pdd* undergoes a stress response at 37°C, with heat-shock proteins accounting for the most upregulated functions at this temperature. On the contrary, *Vibrio* species pathogenic for humans and marine animals as *Vibrio parahaemolyticus*, *V. vulnificus* and *V. cholerae* are routinely cultured at 37°C because such temperature does not represent a limiting factor. Indeed, *V. parahaemolyticus* shows the highest percentage of genes with stable expression at 37°C (Urmersbach et al., 2015).

Growth at 37°C caused the downregulation of most of the virulence-related functions known in *Pdd*. This not only pertains to the *Pdd* cytotoxins, but also to iron acquisition systems, motility and chemotaxis. Previous studies have reported the importance of iron acquisition in *Pdd* virulence for fish and mammals (Fouz et al., 1994, 1997). In the present study, it was demonstrated that growth at 37°C caused downregulation of iron ABC transporters and siderophore receptors. Downregulated genes included VDA_000794 encoding a TonB-dependent siderophore receptor. Of note, this gene was found to be induced when *Pdd* is cultured under iron-limitation conditions (Puentes et al., 2017).

A previous study that compared the *Pdd* transcriptomes at 15 and 25°C, revealed that warm seawater temperatures (25°C) upregulated genes for chemotaxis, flagellar motility and virulence with respect to 15°C (Matanza and Osorio, 2018). This clearly suggests that the optimal temperature for *Pdd* to trigger a virulence expression profile is close to 25°C. Proteins for prevention of oxidative damage, found among the most up-regulated genes at 37°C, are produced by bacteria as defensive strategies against host defenses, and constitute interesting targets for the design of antimicrobial compounds (Ezraty et al., 2017). In this line, another gene upregulated at 37°C was an outer membrane protein (VDA_000104) whose homologs in *V. cholerae* (VC1835) and *V. parahaemolyticus* (VP1390) are related to virulence and antimicrobial activities (Bina et al., 2003; Ronholm et al., 2016). Some of the most DEGs genes unveiled in this study still await further investigation. It is the case of VDA_000412, a protein of the patatin-like phospholipase superfamily that exhibits a 10-fold upregulation at 37°C. The patatin-like ExoU plays an important role in pathogenesis in *Pseudomonas aeruginosa* (Sato and Frank, 2014). Overall, the transcriptomics data reported here are expected to contribute to the investigation of novel approaches for prevention and for less invasive control of human infections caused by *Pdd*. On balance, these results integrate transcriptomics, genomics and phenotypic data providing strong evidence that human infections caused by *Pdd* are opportunistic rather than originated by specific clones adapted to the human host. Transcriptomics analysis reveals that, under human host temperature conditions, *Pdd* triggers a defensive strategy by upregulating heat-shock proteins and defense mechanisms, while downregulating a virulence expression profile.

## Data Availability Statement

The datasets generated for this study can be found in the GenBank: VZUQ00000000 and WAEO00000000.

## Author Contributions

XM and CO conceived the study, designed the experiments, analyzed the data, and wrote the manuscript. XM performed the experiments. All authors contributed to the article and approved the submitted version.

## Conflict of Interest

The authors declare that the research was conducted in the absence of any commercial or financial relationships that could be construed as a potential conflict of interest.

## Footnotes

1 https://www.ncbi.nlm.nih.gov/genome/675

2 http://www.bioinformatics.babraham.ac.uk/projects/fastqc/

3 http://broadinstitute.github.io/picard/
