# Supplementary material for: Exposure of the Opportunistic Marine Pathogen Photobacterium damselae subsp. damselae to Human Body Temperature Is a Stressful Condition That Shapes the Transcriptome, Viability, Cell Morphology, and Virulence
Source: Front Microbiol. 2020 Jul 24;11:1771. doi: 10.3389/fmicb.2020.01771 (PMC7396505; doi:10.3389/fmicb.2020.01771)
Supplement: Supplementary file 1 [file Table_1.DOCX]

**Supplementary Table 1:** Results of the Fatty Acid Methyl Ester (FAME) Analysis of the *Photobacterium damselae* subsp. *damselae* highly virulent strain RM-71, cultivated at 37ºC (top panel) and at 25ºC (Bottom panel).

**TOP PANEL: Results at 37ºC**

| **RT** | **Response** | **Ar/Ht** | **Rfact** | **ECL** | **Peak Name** | **Percent** | **Comment1** | **Comment2** |
| --- | --- | --- | --- | --- | --- | --- | --- | --- |
| 1.608 | 4.18E+08 | 0.026 | ---- | 7.024 | SOLVENT PEAK | ---- | < min rt |  |
| 2.026 | 213 | 0.022 | ---- | 7.876 |  | ---- | < min rt |  |
| 2.178 | 217 | 0.026 | ---- | 8.186 |  | ---- | < min rt |  |
| 3.692 | 546 | 0.037 | 1.124 | 10.924 | Sum In Feature 2 | 0.14 | ECL deviates -0.004 | unknown 10.928 |
| 4.124 | 238 | 0.029 | 1.091 | 11.424 | 10:0 3OH | 0.06 | ECL deviates 0.002 |  |
| 4.642 | 16014 | 0.031 | 1.057 | 12 | 12:0 | 3.76 | ECL deviates 0.000 | Reference -0.004 |
| 5.136 | 788 | 0.029 | 1.034 | 12.438 | 11:0 3OH | 0.18 | ECL deviates 0.000 |  |
| 5.19 | 2203 | 0.033 | 1.032 | 12.486 | unknown 12.484 | ---- | ECL deviates 0.002 |  |
| 5.771 | 375 | 0.031 | 1.009 | 13 | 13:0 | 0.08 | ECL deviates 0.000 | Reference -0.003 |
| 6.017 | 528 | 0.034 | 1.002 | 13.184 | 12:0 2OH | 0.12 | ECL deviates 0.007 |  |
| 6.158 | 386 | 0.333 | 0.998 | 13.288 | 12:1 3OH | 0.09 | ECL deviates 0.000 |  |
| 6.384 | 14135 | 0.034 | 0.992 | 13.455 | 12:0 3OH | 3.16 | ECL deviates 0.001 |  |
| 6.867 | 2956 | 0.035 | ---- | 13.814 |  | ---- |  |  |
| 7.118 | 23851 | 0.037 | 0.974 | 14 | 14:0 | 5.17 | ECL deviates 0.000 | Reference -0.003 |
| 7.835 | 1614 | 0.041 | 0.961 | 14.472 | Sum In Feature 1 | 0.34 | ECL deviates -0.006 | 13:0 3OH/15:1 i H |
| 8.324 | 668 | 0.038 | 0.953 | 14.793 | 15:1 w8c | 0.14 | ECL deviates 0.000 |  |
| 8.639 | 6557 | 0.038 | 0.949 | 15.001 | 15:0 | ---- | ECL deviates 0.001 |  |
| 9.279 | 753 | 0.043 | 0.942 | 15.389 | 16:1 w7c alcohol | 0.16 | ECL deviates 0.002 |  |
| 9.442 | 18604 | 0.042 | 0.94 | 15.487 | Sum In Feature 2 | 3.89 | ECL deviates -0.001 | 14:0 3OH/16:1 iso I |
| 9.55 | 1444 | 0.045 | 0.939 | 15.553 | 16:0 N alcohol | 0.3 | ECL deviates 0.003 |  |
| 9.671 | 380 | 0.033 | 0.938 | 15.626 | 16:0 iso | 0.08 | ECL deviates -0.001 | Reference -0.004 |
| 9.989 | 157247 | 0.042 | 0.935 | 15.819 | Sum In Feature 3 | 32.68 | ECL deviates -0.003 | 16:1 w7c/16:1 w6c |
| 10.289 | 130142 | 0.042 | 0.932 | 16.001 | 16:0 | 26.97 | ECL deviates 0.001 | Reference -0.003 |
| 10.454 | 733 | 0.035 | ---- | 16.097 |  | ---- |  |  |
| 11.648 | 3337 | 0.042 | 0.923 | 16.791 | 17:1 w8c | 0.68 | ECL deviates -0.001 |  |
| 11.771 | 1660 | 0.046 | 0.922 | 16.683 | 17:1 w6c | 0.34 | ECL deviates 0.003 |  |
| 12.007 | 11918 | 0.043 | 0.921 | 17 | 17:0 | 2.44 | ECL deviates 0.000 | Reference -0.004 |
| 13.352 | 2240 | 0.055 | 0.915 | 17.769 | 18:1 w9c | 0.46 | ECL deviates 0.000 |  |
| 13.447 | 61942 | 0.042 | 0.914 | 17.824 | Sum In Feature 8 | 12.59 | ECL deviates 0.001 | 18:1 w7c |
| 13.617 | 956 | 0.043 | 0.914 | 17.921 | 18:1 w5c | 0.19 | ECL deviates 0.002 |  |
| 13.775 | 18414 | 0.042 | 0.913 | 18 | 18:0 | 3.74 | ECL deviates 0.000 | Reference -0.005 |
| 13.899 | 8459 | 0.045 | 0.913 | 18.082 | 18:1 w7c 11-methyl | 1.72 | ECL deviates 0.001 |  |
| 15.228 | 726 | 0.044 | 0.908 | 18.847 | Sum In Feature 7 | 0.15 | ECL deviates 0.001 | un 18.846/19:1 w6c |
| 15.335 | 889 | 0.049 | 0.907 | 18.908 | 19:0 cyclo w8c | 0.18 | ECL deviates 0.006 |  |
| 16.723 | 540 | 0.041 | ---- | 19.708 |  | ---- |  |  |
| 16.936 | 1001 | 0.044 | 0.901 | 19.381 | 20:1 w7c | 0.2 | ECL deviates 0.000 |  |
| 18.365 | 736 | 0.038 | ---- | 20.657 |  | ---- | > max rt |  |
| ---- | 1614 | ---- | ---- | ---- | Summed Feature 1 | 0.34 | 15:1 iso H/13:0 3OH | 13:0 3OH/15:1 i H |
| ---- | 19150 | ---- | ---- | ---- | Summed Feature 2 | 4.02 | 12:0 aldehyde | unknown 10.928 |
| ---- | ---- | ---- | ---- | ---- |  | ---- | 16:1 iso I/I4:0 3OH | 14:0 3OH/16:1 iso I |
| ---- | 157247 | ---- | ---- | ---- | Summed Feature 3 | 32.68 | 16:1 w7c/16:1 w6c | 16:1 w6c/16:1 w7c |
| ---- | 726 | ---- | ---- | ---- | Summed Feature 7 | 0.15 | un 18:846/19:1 w6c | 19:1 w6c/846/19cy |
| ---- | ---- | ---- | ---- | ---- |  | ---- | 19:0 cyclo w10c/19w6 |  |
| ---- | 61942 | ---- | ---- | ---- | Summed Feature 8 | 12.59 | 18:1 w7c | 18:1 w6c |

**BOTTOM PANEL: Results at 25ºC**

| **RT** | **Response** | **Ar/Ht** | **Rfact** | **ECL** | **Peak Name** | **Percent** | **Comment1** | **Comment2** |
| --- | --- | --- | --- | --- | --- | --- | --- | --- |
| 1.608 | 4.10E+08 | 0.025 | ---- | 7.025 | SOLVENT PEAK | ---- | < min rt |  |
| 2.026 | 168 | 0.021 | ---- | 7.877 |  | ---- | < min rt |  |
| 2.179 | 153 | 0.023 | ---- | 8.189 |  | ---- | < min rt |  |
| 3.692 | 391 | 0.031 | 1.124 | 10.923 | Sum In Feature 2 | 0.09 | ECL deviates -0.005 | unknown 10.928 |
| 4.123 | 305 | 0.031 | 1.091 | 11.422 | 10:0 3OH | 0.07 | ECL deviates 0.000 |  |
| 4.643 | 14498 | 0.031 | 1.057 | 12 | 12:0 | 3.16 | ECL deviates 0.000 | Reference -0.004 |
| 5.137 | 795 | 0.031 | 1.034 | 12.439 | 11:0 3OH | 0.17 | ECL deviates 0.001 |  |
| 5.19 | 1612 | 0.032 | 1.032 | 12.486 | unknown 12.484 | ---- | ECL deviates 0.002 |  |
| 5.771 | 248 | 0.029 | 1.009 | 13.000 | 13:0 | 0.05 | ECL deviates 0.000 | Reference -0.004 |
| 6.015 | 475 | 0.033 | 1.002 | 13.182 | 12:0 2OH | 0.1 | ECL deviates 0.005 |  |
| 6.383 | 12612 | 0.035 | 0.992 | 13.455 | 12:0 3OH | 2.58 | ECL deviates 0.001 |  |
| 6.867 | 2369 | 0.035 | ---- | 13.814 |  | ---- |  |  |
| 7.117 | 15930 | 0.037 | 0.974 | 14 | 14:0 | 3.2 | ECL deviates 0.000 | Reference -0.003 |
| 7.835 | 866 | 0.042 | 0.961 | 14.472 | Sum In Feature 1 | 0.17 | ECL deviates 0.006 | 13:0 3OH/15:1 i H |
| 8.324 | 687 | 0.037 | 0.953 | 14.793 | 15:1 w8c | 0.13 | ECL deviates 0.000 |  |
| 8.64 | 3534 | 0.039 | 0.949 | 15.001 | 15:0 | ---- | ECL deviates 0.001 |  |
| 9.095 | 411 | 0.032 | ---- | 15.277 |  | ---- |  |  |
| 9.445 | 13774 | 0.041 | 0.94 | 15.489 | Sum In Feature 2 | 2.67 | ECL deviates 0.001 | 14:0 3OH/16:1 iso I |
| 9.991 | 201624 | 0.042 | 0.935 | 15.82 | Sum In Feature 3 | 38.83 | ECL deviates -0.002 | 16:1 w7c/16:1 w6c |
| 10.138 | 1545 | 0.048 | 0.933 | 15.909 | 16:1 w5c | 0.3 | ECL deviates 0.000 |  |
| 10.29 | 91613 | 0.04 | 0.932 | 16.001 | 16:0 | 17.6 | ECL deviates 0.001 | Reference -0.002 |
| 11.65 | 3687 | 0.042 | 0.923 | 16.792 | 17:1 w8c | 0.7 | ECL deviates 0.000 |  |
| 11.771 | 1770 | 0.044 | 0.922 | 16.862 | 17:1 w6c | 0.34 | ECL deviates 0.002 |  |
| 12.008 | 4829 | 0.043 | 0.921 | 17 | 17:0 | 0.92 | ECL deviates 0.000 | Reference -0.003 |
| 13.356 | 1999 | 0.058 | 0.915 | 17.77 | 18:1 w9c | 0.38 | ECL deviates 0.001 |  |
| 13.451 | 136741 | 0.043 | 0.914 | 17.825 | Sum In Feature 8 | 25.76 | ECL deviates 0.002 | 18:1 w7c |
| 13.617 | 1355 | 0.053 | 0.914 | 17.919 | 18:1 w5c | 0.26 | ECL deviates 0.000 |  |
| 13.758 | 9068 | 0.044 | 0.913 | 18 | 18:0 | 1.71 | ECL deviates 0.000 | Reference -0.003 |
| 13.898 | 3602 | 0.043 | 0.913 | 18.08 | 18:1 w7c 11-methyl | 0.68 | ECL deviates -0.001 |  |
| 16.72 | 473 | 0.044 | ---- | 19.705 |  | ---- |  |  |
| 16.94 | 862 | 0.042 | 0.901 | 19.832 | 20:1 w7c | 0.16 | ECL deviates 0.001 |  |
| 18.364 | 573 | 0.035 | ---- | 20.655 |  | ---- | > max rt |  |
| ---- | 866 | ---- | ---- | ---- | Summed In Feature 1 | 0.17 | 15:1 iso H/13:0 3OH | 13:0 3OH/15:1 i H |
| ---- | 14165 | ---- | ---- | ---- | Summed In Feature 2 | 2.76 | 12:0 aldehyde | unknown 10.928 |
| ---- | ---- | ---- | ---- | ---- |  | ---- | 16:1 iso I/I14:0 3:OH | 14:0 3OH/16:1 iso I |
| ---- | 201624 | ---- | ---- | ---- | Summed In Feature 3 | 38.83 | 16:1 w7c/16:1 w6c | 16:1 w6c/16:1 w7c |
| ---- | 136741 | ---- | ---- | ---- | Summed In Feature 8 | 25.76 | 18:1 w7c | 18:1 w6c |
